# Rare Complication of non-Treated Abdominal Aortic Aneurysm: Extensive Thrombus in Right Cardiac Chambers

**DOI:** 10.5935/abc.20160143

**Published:** 2016-10

**Authors:** Viviane Tiemi Hotta, David A. Bluemke, Kamila Fernanda Staszko, Ana Neri Rodrigues Epitacio Pereira, Carlos Eduardo Rochitte

**Affiliations:** 1 Instituto do Coração - Faculdade de Medicina da Universidade de São Paulo, São Paulo - Brazil; 2 National Institutes of Health Clinical Center, Bethesda - USA

**Keywords:** Aortic Aneurysm, Abdominal / complications, Thrombosis, Heart Atria, Echocardiography

## Abstract

A 78-year-old patient presented with shortness of breath after falling down. Transthoracic echocardiogram showed an extensive thrombus in the right atrium (RA), extensive thrombosis of the inferior vena cava (IVC), and abdominal aortic aneurysm (AAA). A magnetic resonance confirmed the thrombosis of the RA extending to the IVC, which was apparently fused to the abdominal aortic aneurysm (compression? erosion?). This case illustrates a severe and rare complication of a non-treated AAA. There probably was IVC erosion by the aortic aneurysm, leading to blood stasis and extensive thrombosis of the IVC and right cardiac chambers.

## Case Report

**Figure 1:**
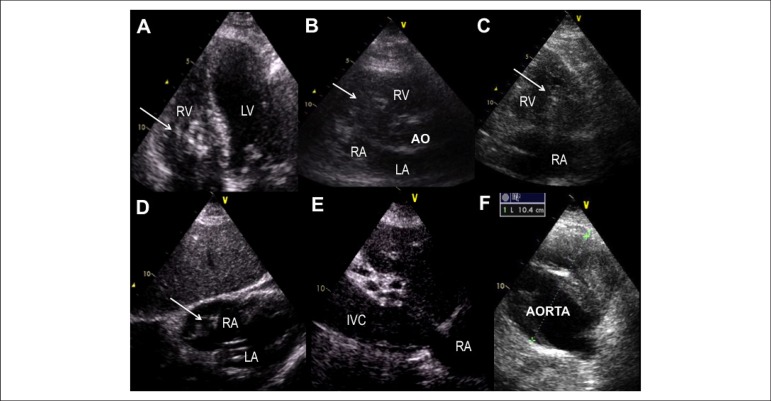
Images from transthoracic echocardiography. Large dimension thrombus (arrows) in right cardiac chambers depicted on apical four-chamber view (A), parasternal short-axis view of the basal right ventricle (B), parasternal long-axis view of the right ventricle inflow (C) and subcostal view (D). Extensive thrombosis of the inferior vena cava (E) and an aortic aneurysm with superimposed thrombosis (F) on subcostal view. Ao: Aorta; IVC: Inferior vena cava; LA: Left atrium; LV: Left ventricle; RA: Right atrium; RV: Right ventricle.

**Figure 2:**
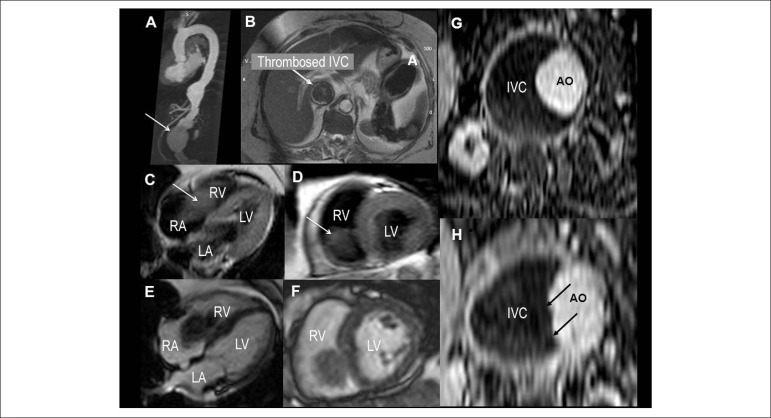
Cardiac Magnetic Resonance Images. (A) Sagittal view of thoracic and abdominal magnetic resonance showing the abdominal aortic aneurysm (arrow), (B) extensive thrombosis of the IVC on a transverse view (arrow) of a SSFP image (Steady State Free Precession) (T2 weighted). Double IR (inversion recovery) – FSE (Fast Spin Echo) in Four-Chamber (C) and Short axis (D) views showing a large thrombus in the right cardiac chambers. LGE (Late gadolinium enhancement) images in Four-Chamber (E) and short axis (F) views showing the large thrombus. Axial Images superior (G) and inferior (H) of abdominal magnetic resonance of a SSFP image. Double IR – FSE. On H, the arrows show the irregular and probably eroded aortic aneurysm into the IVC. IVC: Inferior vena cava; AO: Aorta; LA: Left atrium; LV: Left ventricle; RA: Right atrium; RV: Right ventricle.

A 78-year-old female patient presented with shortness of breath after falling down. She reported a history of systemic hypertension, stable coronary artery disease and abdominal aorta aneurysm undergoing clinical surveillance. The laboratory exams evidenced thrombocytopenia (22.000 mm^3^) with no other abnormalities. Transthoracic echocardiogram showed a heterogeneous hyper-echogenic mass at the right atrium, protruding into the right ventricle, highly mobile, suggestive of thrombus.^1-3^ Extensive thrombosis of the inferior vena cava (IVC) (Figure 1), and abdominal aorta aneurysm (AAA) of around 10 cm in diameter was also observed. The patient underwent thoracic, abdominal and pelvic computed tomography (CT). CT evidenced irregular, tortuous supra and infra-renal aortic aneurysm. There were paravertebral as well as ventral abdominal wall collaterals, indicating venous obstruction and signs of probable erosion of the IVC by the aneurysm. An aortic magnetic resonance was performed to better evaluate the aorta anatomy, which confirmed the extensive thrombosis of the IVC, in the segment related to the AAA, with extension of the thrombosis to the right cardiac chambers (Figure 2). Anticoagulation therapy was contraindicated due to the thrombocytopenia and a discrete increase in platelet count was observed after corticosteroid and immunoglobulin therapy. The patient was discharged on request and died of sudden death ten days after dismissal.

Right atrial masses are very rare findings. This case illustrates a severe and rare complication of a non-treated AAA. Enteric erosion is a well-known complication of aortic aneurysm or aortic dissection. Arteriovenous fistula has been described as a graft-related complication after AAA repair,^4^ but to the best of our knowledge, this has not been reported for the native aorta and IVC. Unfortunately, this diagnosis was suspected in this patient by means of cardiovascular imaging techniques but not confirmed by anatomical pathological analysis.

The video is available online: Movie clip for Figure 1A.

**Video:** Access the video through the link: http://www.arquivosonline.com.br/2016/english/10704/video_ing.asp RV: right ventricle; LV: left ventricle; RA: right atrium; LA: left atrium.
